# A participatory approach to model the neighbourhood food environment

**DOI:** 10.1371/journal.pone.0292700

**Published:** 2024-01-24

**Authors:** Amanda Karapici, Steven Cummins

**Affiliations:** 1 Department of Public Health, Environments & Society, London School of Hygiene & Tropical Medicine, London, United Kingdom; 2 Professor of Population Health & NIHR Senior Investigator, Department of Public Health, Environments & Society, London School of Hygiene & Tropical Medicine, London, United Kingdom; Federal University of Minas Gerais: Universidade Federal de Minas Gerais, BRAZIL

## Abstract

Inequalities in exposure to a health-promoting local food environment have been implicated in the generation of inequalities in diet-related behaviours and outcomes, including obesity and diabetes. Increasingly, poor diet and diet-related disease have been characterised as an emergent property of a complex system and, as such, the drivers of poor diet may be better understood by using a complex system perspective. In this study, we describe a participatory approach for understanding the system drivers of unhealthy food consumption. System dynamics (SD) was used to identify, understand, and visualise the elements of the neighbourhood food retail system that influence individuals’ eating behaviour. Group Model Building (GMB), undertaken online with stakeholders (n = 11), was used to funnel existing knowledge and evidence on urban food environments and to build a conceptual system map of the local food retail environment inclusive of the drivers that influence the decision to purchase and consume meals that are high in fat, salt, and sugar (HFSS), and calories. The GMB was organised as a knowledge elicitation process involving a questionnaire, a workbook, and a structured workshop. The GMB generated a comprehensive causal loop diagram (CLD) of the retail environment inclusive of the drivers that influence the decision to purchase and consume unhealthy meals. The CLD was designed around two main variables (i) exposure to food outlets and (ii) food consumption. The system map built during the Group Model Building session linked exposure to food outlets with the possibility to purchase and consume unhealthy meals. The effectiveness of this link will be tested in an Agent-Based model. The conceptual model illustrates the complexity of the factors responsible for inequalities in unhealthy eating. The GMB approach provides a basis for building an agent-based model for local authorities to characterise their food retail environment, uncover potential leverage points for interventions and test them ‘in silico’ in a virtual environment.

## Introduction

Dietary habits play a significant role in weight gain and maintenance, and the food environment is seen as an increasingly important determinant of dietary habits. Although individual-level determinants of diet, such as age, gender, ethnicity, income, education, household composition, taste, nutrition knowledge and food preparation skills are important, diet is also influenced by the environment in which we live. Food environments have received recognition for their importance in enhancing or damaging dietary habits by shaping individual behaviour. A large and expanding evidence base has primarily focused on the neighbourhood food environment and the presence/absence of supermarkets, grocery and convenience stores, fast-food outlets, and restaurants. The evidence also looks at the density of these stores and their distance from one another, as well as the in-store price and availability of food. Several studies [1–4] suggest that these factors are potentially modifiable through policy intervention. Evidence suggests that food environments and dietary habits are linked but understanding the true causal effect of the food environment on diet and obesity poses methodological challenges.

The consumption of energy-dense and ultra-processed is believed to be an important contributor to the increased risk of obesity, with many of these foods primarily associated with out-of-home consumption [5]. Eating food prepared out-of-home is becoming increasingly common and is a growing contributor to an individual’s total energy intake and household spending on food [5–7]. In the UK, eating out-of-home has undergone considerable growth over the past decade. Data from 2019–20, shows that UK households spend an average of £19.50 per week on restaurant and café meals, £5.60 on takeaways and snack food eaten outside the home, and £5.40 on takeaways eaten at home [8] showing a 4.72% increase on restaurant and café meals spending and a 9.34% increase in spending on snacks compared to the financial year 2017/18 [9].

As our understanding of the aetiological complexity of obesity has progressed [10], the totality of factors that affect individual food choice remain difficult to assess as individuals may have different motivations, preferences, and decision-making processes. Individuals’ choice of food is influenced by a complex interplay of factors including personal preference, genetics, cultural and social norms, availability, income, education, and more. While the role of individual risk and genetic factors has been well-researched, the interactions between individual-level and environmental factors remain less clear.

Factors that affect individual food choices may be nonlinear, difficult to distinguish from individuals’ attributes and also interact with each other and the environment over time [11, 12]. For these reasons, standard epidemiological approaches fail to fully capture these relationships as they struggle to incorporate dynamic elements, such as feedback and adaptations resulting from interactions between people and their environment. Hence, systems science methods are seen as potentially useful tools for examining these complex dynamic processes.

System Dynamics has a long history of facilitating the learning process of complex systems [13]. Group Model Building is an established practice within SD, that captures the knowledge of stakeholders and translates it into informal map models called Causal Loop Diagrams. According to Forrester [14], experts draw on their mental database of various decision-making points in a system, making it a reliable source of information when dealing with organisations and political structures when designing policy. This information is therefore a fundamental data source for model building and designing policy.

This study is part of a larger research project that aims to better understand the role of the neighbourhood food retail environment in the consumption of unhealthy food purchased outside of home. The project leverages the power of two methodologies, Group Model Building, and Agent-Based Modelling, to achieve these goals. This paper presents the findings from the first stage of this project. Group Model Building was utilized to gather information on the neighbourhood food retail environment and identify the key variables that impact the purchasing and consumption of high-fat, high-sugar foods consumed outside of the home. A Causal Loop Diagram of the neighbourhood food retail environment was then created. This method was selected because it has been widely used in systems dynamics and has been proven to effectively capture the knowledge of stakeholders.

## Methodology

### Ethics

This study was approved by the London School of Hygiene and Tropical Medicine Ethics Committee (ref: 22779–1). Participation was voluntary and all responses were anonymised. Participants provided written informed consent to participate in this study.

### Study design

**Fig 1:**
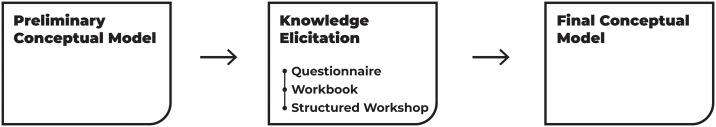
Knowledge elicitation process [adapted from [15, 16]], integrated within the GMB.

Group Model Building was used to create a system map of the neighbourhood food environment and its influence on eating behaviour. Given fieldwork restrictions related to the pandemic, the GMB was developed in an online environment and consisted of three stages: (i) creation of a preliminary conceptual model, (ii) knowledge elicitation from a stakeholder group, and (iii) development of a final system model. The knowledge elicitation process incorporated within the GMB was adapted from Vennix and Gubbels [15, 16] (Fig 1). The process, tailored to the iterative nature of the model building, was designed to allow a structured discussion of the participants’ views about the real world within a reasonable timeframe. Data collection occurred in the knowledge elicitation process.

### Preliminary conceptual model

The goal was to create a basic conceptual model that could accurately depict the important factors related to the local food retail environment. During the literature review phase, it was decided to build a preliminary model as a starting point instead of creating a model from scratch with the stakeholders. This approach was chosen because there is already a substantial amount of existing evidence available, and it also helps to accelerate the model building process [16]. In addition, it facilitated discussion and helped explain tasks to practitioners and academics who were not familiar with SD.

The preliminary model was conceptualised by the modeller by interpreting and analysing relevant written statements during the literature review and creating causal links from the text, a practice adopted from Axelrod [17]. The modeller’s thorough analysis of the written statements found in the literature led to the identification of potential variables and their underlying relationships. In cases where appropriate, thus when evidence suggested that changes in one variable directly caused changes in another variable, these relationships were converted into causal links, and served as the basis for the preliminary model. Next, the causal relationship was checked again against the relevant literature, in order to see if the representation was represented correctly. Nevertheless, no effort was made at this point to build a complete model since the objective was to provide a base model which aimed to stimulate a structured debate over the components of the system during the knowledge elicitation process.

### Knowledge elicitation process

#### Sampling and recruitment

There is no ideal number of participants in Group Model Building [18]. When dealing with policy-oriented modelling, the modeller is faced with several dilemmas regarding group size and group diversity. The aim when selecting participants is to capture a variety of viewpoints to ensure that the model will not become individualistic [14, 19, 20]. Group size should strike a balance as increasing the group size and diversity might be beneficial to the quality of the model but it may also produce greater friction among the group, lowering group performance [18].

The identification of participants and recruitment process started in April 2021. Participants were identified based on their field of expertise and what we knew about their technical knowledge. The focus was on involving individuals that had information that we couldn’t gain elsewhere. Thus, recruited individuals had extensive knowledge about the topic and rich mental models that needed to be captured. To include a variety of viewpoints we recruited both researchers and practitioners. Researchers had theoretical knowledge about diet, food systems and individual behaviour and practitioners were experts from scientific, government, and public institutions who had hands-on policy and practice-based knowledge in dealing with unhealthy food environments (Table 1). The selection of participants was done using snowball sampling. The research team first asked one professor engaged in this research to recommend an initial pool of academics and practitioners. Then, the same inquiry was made to other potential participants identified from the initial pool. The recommended names were crosschecked among the different lists and the final list of individuals was drafted.

**Table 1:** Profiles of GMB participants.

*CATEGORY*	*FREQUENCY*	*PERCENTAGE*
***Age***:		
*<25 years*	0	0
*26 to 35 years*	2	18
*36 to 45 years*	4	36.5
*46 to 55 years*	4	36.5
*56 to 65 years*	1	9
*>65 years*	0	0
***Gender***		
*Female*	7	63
*Male*	4	37
***Participants’ classification***:		
*Researchers*	6	54.5
*Decision Makers*	1	9
*Practitioners*	4	36.5
***Total***	11	100

**Fig 2:**
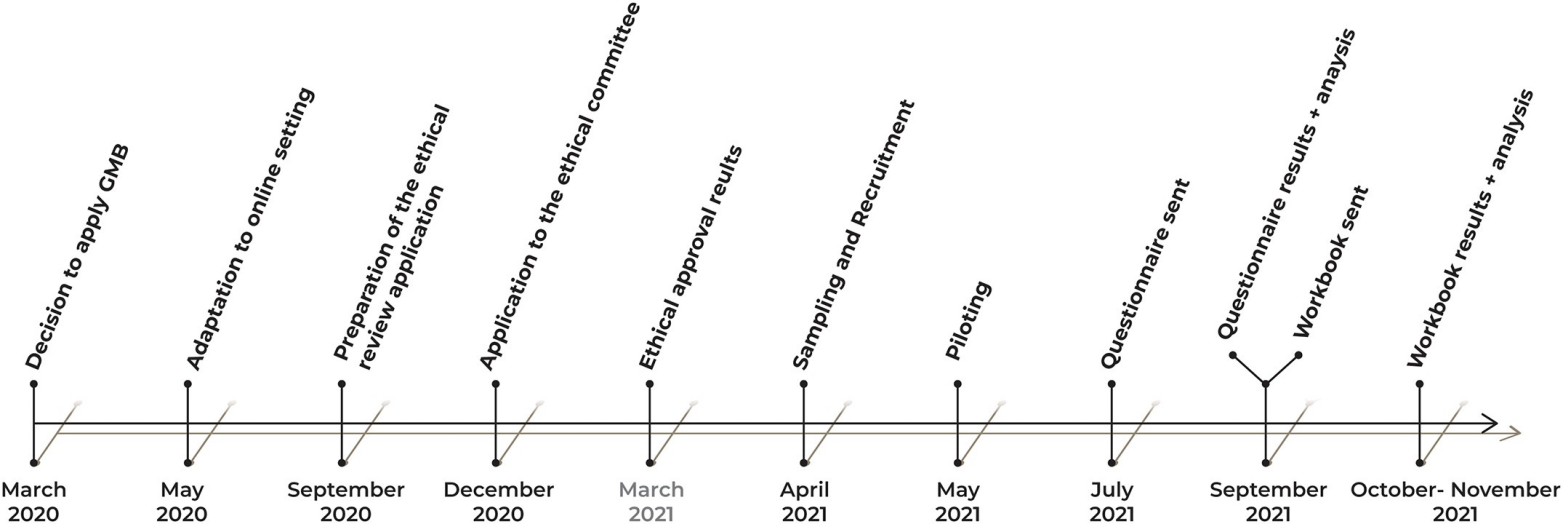
Timeline of GMB.

We invited twenty-six participants to participate in the research (Fig 2), and nineteen agreed to do so. Eleven filled in both the questionnaire and workbook during the knowledge elicitation process.

#### Stage 1—The questionnaire

**Fig 3:**
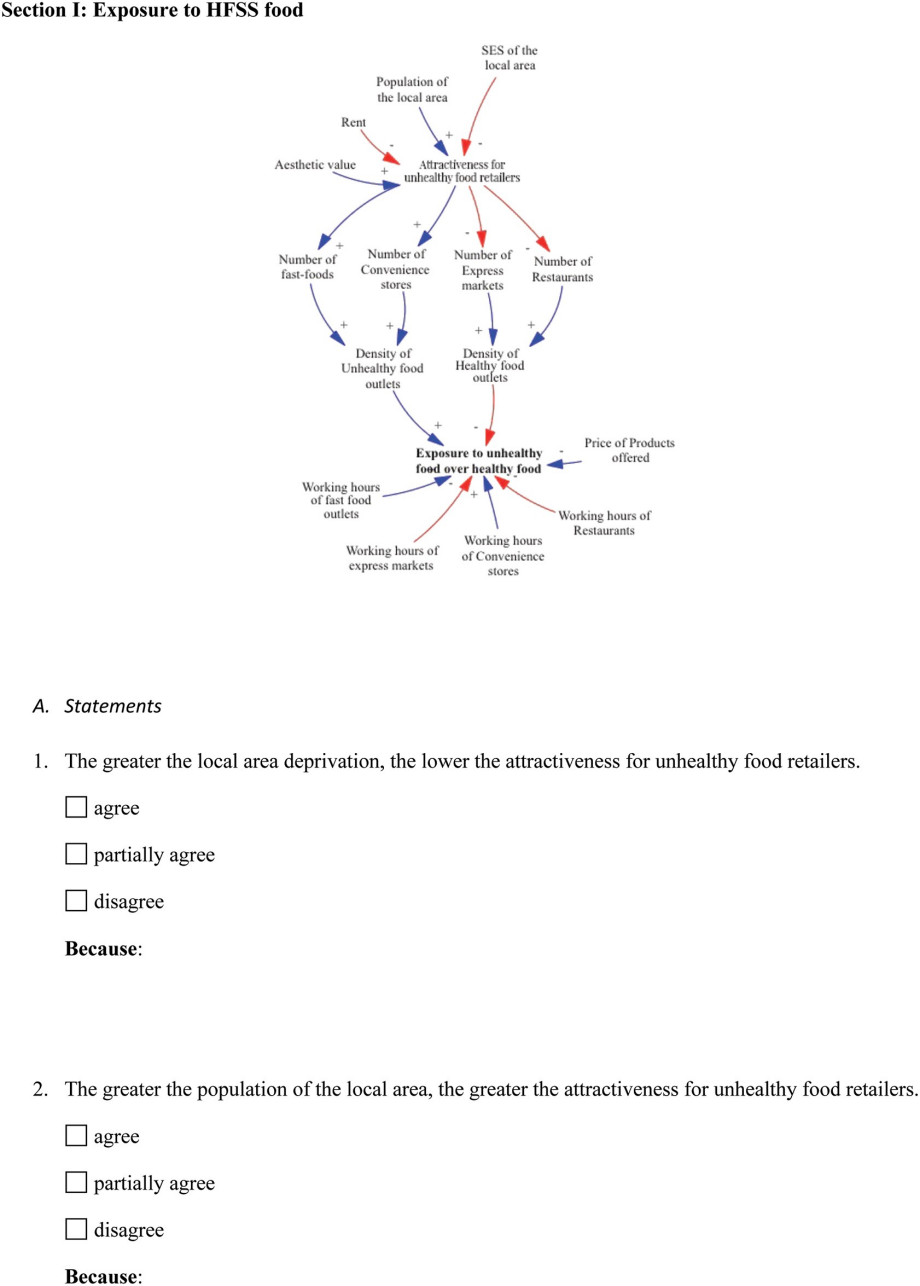
Snapshot from the questionnaire.

The knowledge elicitation process had three stages. Stage 1 comprised a questionnaire that was sent via e-mail to participants asking for their expert opinion about the preliminary conceptual model (Fig 3). The questionnaire was 10 pages in length and required an average of 18 minutes to be completed.

The questionnaire began by defining the project title and the purpose of the questionnaire and continued by explaining the purpose of the research and the problem under investigation. Next, the preliminary conceptual model was presented to the participants and the goals of the tasks were made explicit to them. The remainder of the questionnaire was divided into three sections, each dealing with one dependent variable.

Sections one and two introduced the CLD relevant to the dependent variable, followed by statements indicating the causal relationship between the dependent variables and other variables. Here, participants were asked if they agreed, partially agreed, or disagreed with the given statement and why. At the end of these sections, the participants were asked to add additional parameters that affected the main variable, which were not considered in the preliminary model. To eliminate the risk of developing a large list of variables, participants were asked to indicate the three most important factors. Section three aimed to capture the mental models of the participants and see how they would link the two main variables presented in the previous sections.

The questionnaire was piloted to explore the validity of its statements, the clarity of the tasks to be completed by participants, and to assess what was a reasonable length of time to fill in the questionnaire to maximise response. Pilot testing enabled us to detect and correct unclear questions and instructions. In addition, the feasibility and practicality of the questionnaire, including the length and time required to complete it, were tested. As a result, valuable information was gathered through this process that informed further improvement to the questionnaire and assured an overall improvement in the reliability and validity of it, resulting in a more useful and higher-quality instrument.

#### Stage 2—The workbook

**Fig 4:**
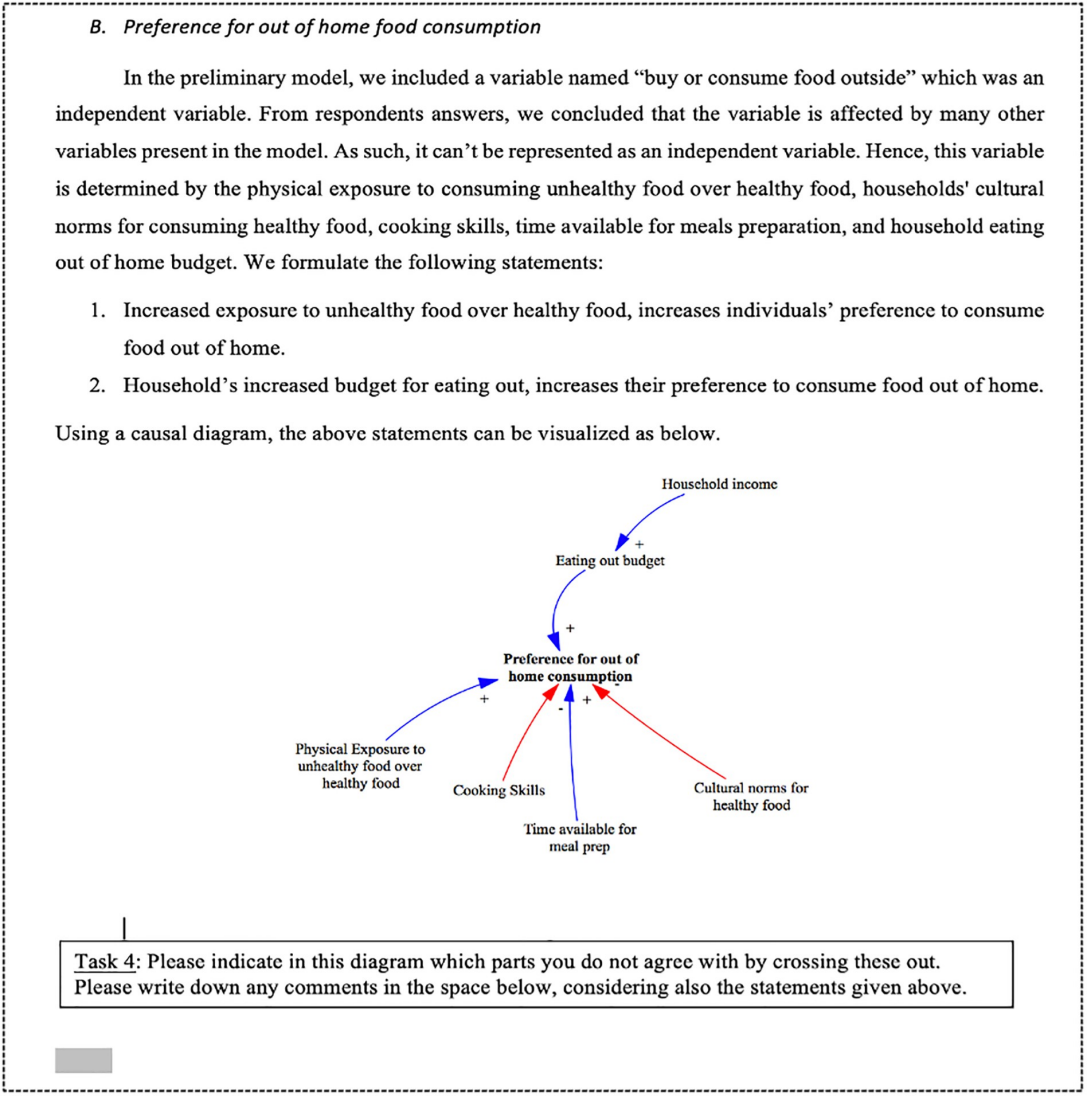
Snapshot from the workbook.

Stage 2 of the knowledge elicitation continued with the drafting of the workbook (Fig 4). The goal of the workbook was to address any last uncertainties from the questionnaire, validate the statements formulated by the participants and prepare the participants for the workshop. The content of the workbook was developed using the information obtained from the questionnaire responses and followed the same structure as the questionnaire. Each section started with a general discussion of the results gathered from the questionnaire for the specified dependent variable—i.e., exposure to HFSS food, and consumption of HFSS food. Different from the questionnaire, the workbook considered a collection of related variables instead of just two-way connections.

To create the workbook, a careful review of the questionnaire was conducted to ensure that the data collected was accurately understood. The review process involved a thorough examination of the questionnaire responses to identify key themes and insights, and to confirm that any relevant information was not overlooked or misinterpreted. This review helped to ensure that the workbook content was grounded in a clear and accurate understanding of the data collected from the questionnaire. Arguments raised in the questionnaire according to each statement were elaborated on and presented in terms of causal links with assigned polarity. Next, the discussion was directed to a narrower concept within the CLD, where the initial concept formulation was introduced, and the participants’ answers were summarised to justify a new representation of the variable. This new representation was communicated initially in written statements describing the relationship of the variable with the main variable, and then they were visually represented in the CLD. Here, participants were asked to comment, criticise, add, or even cross out links and variables presented in the new model version (the model developed after the questionnaire). The new concepts were integrated into the workbook gradually.

As with the questionnaire, the workbook was piloted to understand if the written statements were easy to understand and fill in. The workbook was 14 pages in length and required an average of 25 minutes to be completed.

#### Stage 3—The workshop

Stage 3 of the knowledge elicitation process, the workshop, was conducted in July 2022. The objectives of the workshop were to build a shared understanding of the food environment developed in previous steps, to collect further feedback, identify leverage points in the system and elicit potential policies.

The format of a group model building workshop was adapted to suit the specific needs and constraints of the project. Hence, the workshop was delivered online using the Zoom platform and its whiteboard feature was used, which enabled participants to write their ideas and interact directly with the conceptual model. The workshop was designed in a manner that encouraged active participation from the attendees, as outlined below.

**Fig 5:**
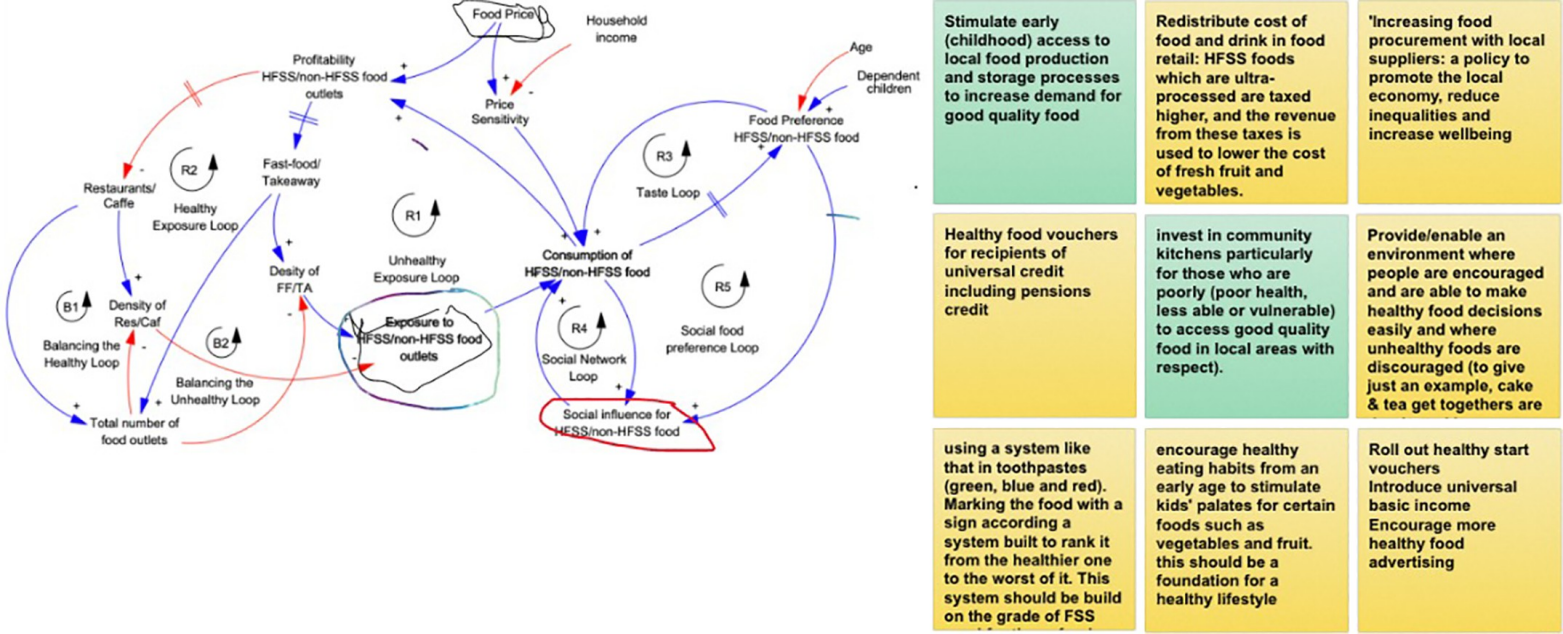
Screenshot of the whiteboard used during the workshop.

The workshop’s agenda encompassed three main components, each contributing to the understanding and elicitation of effective interventions. Fig 5 provides an extract of the whiteboard and participants’ reflections on how to achieve a healthier food environment.

#### Summary of the process

The workshop started with the facilitator introducing the workshop’s goals, objectives, and expectations. Next, a comprehensive summary of the relevant processes and dynamics within the system under study was presented. Participants were provided with a clear overview of the system’s variables, interactions, and key factors influencing its behaviour. This process was aided by using the “Model Review” script from Scriptapedia [21]. The script aims to summarise dynamics insights and clarify any ambiguous concepts while capturing additional information about the model structure. This process served to enhance the shared understanding for all participants, establishing a common starting point for further discussions and analyses. Then, the participants engaged in a thorough group discussion, and debated the model until consensus was reached on key assumptions and relationships. Several similar iterations were done to refine the model in order to generate a clear and complete understanding of relationships.

#### Identify leverage points to intervene in the system

One of the workshop objectives was to identify critical leverage points within the system, where targeted interventions could yield significant and desirable changes. To achieve this objective the “Places to intervene” script was used from Scriptapedia [21]. The script aims to help participants identify potential places for interventions based on two main criteria: feasibility and impact. The GMB participants were asked to identify as many places of intervention as possible within the model that would lower the consumption of HFSS food (Fig 5, left side). Participants were encouraged to consider the feasibility of making changes to each variable and assess the potential impact of those changes by examining connected feedback loops and the number of variables linked to the intervention point.

#### Policy elicitation

In this phase of the workshop, participants engaged in eliciting and proposing policies that could be implemented to leverage the identified leverage points and bring about positive systems change that lowers the purchase and consumption of HFSS food. Drawing upon their expertise and diverse perspectives, participants generated a range of potential policies and discussed their feasibility, potential risks, and expected outcomes (Fig 5, right side). To achieve this goal, the “Initial policy options” from Scriptapedia [21] was used. Although, the objective of this script is to help the group model-building team frame the problem and elicit variables at the beginning of a group model-building session. At this stage, this script was used to generate a list of candidate policy options related to the identified leverage points. Participants collaborated to write short phrases naming policies that they would like to see discussed, modelled and simulated in the agent-based model.

### Final conceptual model

After the workshop, the facilitator refined the final version of the conceptual model. All materials generated during the workshop, such as the facilitator’s notes, observations, and diagrams, were consolidated. Subsequently, the materials were carefully revised, and the model was updated as necessary, incorporating the valuable insights and information collected from the workshop.

## Results

The final number of participants (n = 11), although smaller than anticipated, had good performance in delivering the assigned tasks. The quality of the output met the expectations of the facilitator. Throughout the knowledge elicitation process, the participants worked effectively and efficiently demonstrating good communication and problem-solving skills. As a result, the participants achieved the goals and objectives specified in the Group Model Building process.

### Pilot testing results

Results from the pilot testing of the questionnaire revealed several important findings that warrant careful consideration in the refinement process. Initially, it was projected that the average time required to complete the questionnaire would be approximately 15 minutes. However, the actual time recorded during the piloting phase was longer than expected, with participants taking approximately 20 minutes to complete the questionnaire.

In addition to the time discrepancy, participants provided valuable feedback on various aspects of the questionnaire. One significant observation was the need for improvement in the section describing the purpose of the research. Participants expressed a desire for more detailed information about the research process and a clearer outline of what was expected from them as participants. As a result, revisions were proposed to enhance the purpose section, with the aim of providing a more comprehensive and transparent overview for future participants. To facilitate ease of response and enhance user experience, tick boxes were introduced in the questionnaire template. This simple, yet effective modification significantly improved the process of responding to the statements.

Furthermore, the questionnaire underwent meticulous content review, resulting in the rewriting of certain statements to make them clearer and simpler for participants. This entailed also renaming certain variables that were causing confusion among the participants. To enhance understanding and context, the research team decided to include relevant screenshots of specific parts of CLD. These visual aids were added to aid participants in grasping the concepts and connections within the CLD, potentially improving the overall quality of responses.

Results from the pilot testing of the workbook indicated that the workbook was well-received by the participants, with minimal need for essential changes. The feedback predominantly highlighted formatting issues rather than substantial content or structural concerns. These observations were addressed promptly.

### Preliminary conceptual model

**Fig 6:**
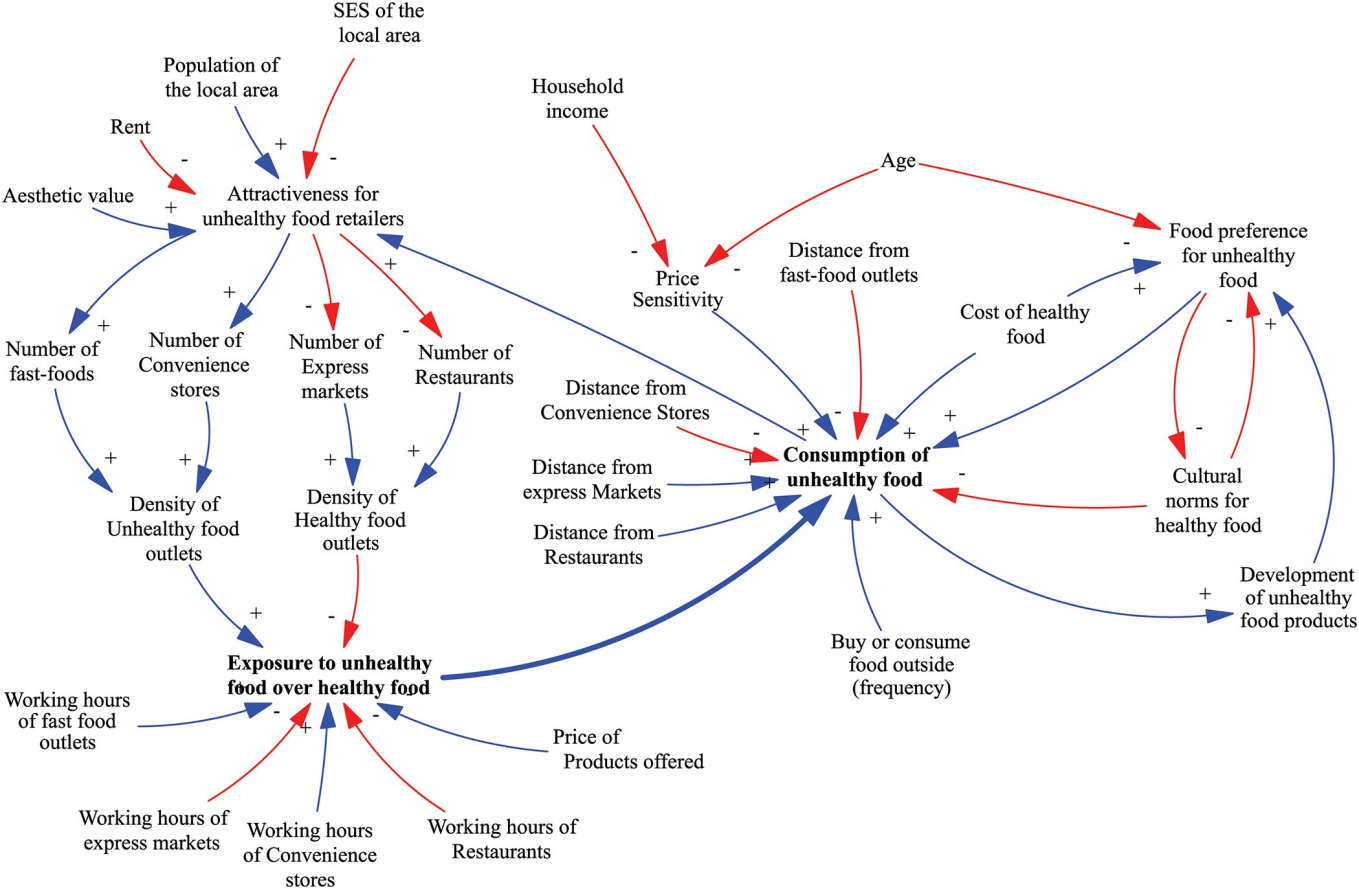
Preliminary conceptual model. Blue line represents a positive link, indicating a move in the same direction. Red line represents a negative link, indicating a move in different directions. Greyed variables represent shadow variables that are defined elsewhere in the model.

The preliminary conceptual model (Fig 6) used in the questionnaire comprised thirty variables divided into two main sections centred around two variables: exposure to HFSS food and consumption of HFSS food. These variables were selected as important based on their relevance to the problem being analysed.

Table 2 provides definitions for the variables present in the final model. This table can be used to understand also the variables included in the preliminary conceptual model. These variables are considered to have a potential impact on the outcome of the study, as they are closely related to our research questions. In the questionnaire, participants indirectly validated the inclusion of these variables in the analysis.

**Table 2:** List of variables and description.

No.	Variable	Description
1.	Degree of urbanisation	A classification that indicates the character of an area
2.	Presence of shopping malls	Number of shopping malls or large retail complexes that often house a variety of shops, restaurants, entertainment venues, and other amenities.
3.	Socioeconomic status (SES) of the local area	The average SES of the people living in that area, determined by the distribution of factors such as income, education, and occupation among the population.
4.	Population of the local area	The number of people residing within a defined geographical region.
5.	Perceived safety of the neighbourhood	Subjective evaluation of individuals’ feelings of security and vulnerability within that specific area. It encompasses the subjective sense of safety and potential risks perceived.
6.	Rent of commercial space	The annual cost of leasing a property for use as a business or commercial space.
7.	Attractiveness for unhealthy food retailers	The degree to which food establishments that sell foods that are high in calories, fat, salt, and sugar are appealing to set up shop in a particular location. This attractiveness is influenced by factors such as the demand for unhealthy food options, SES of the local area, and presence of shopping malls.
8.	Attractiveness for food retailers	The degree to which food establishments in general are appealing to set up shop in a particular location. This attractiveness is influenced by factors such as population of the local area, SES of the local area, perceived safety, and rent of commercial space.
9.	Total number of unhealthy food outlets	The total number of unhealthy food outlets in a specific area refers to the number of food establishments that sell foods that are high in calories, fat, salt, and sugar within a defined geographic region.
10.	Total number of healthy food outlets	The total number of healthy food outlets in a specific area refers to the number of food establishments that sell nutritious, wholesome, and minimally processed foods within a defined geographic region.
11.	Density of unhealthy food outlets	The concentration of unhealthy food outlets in a given location.
12	Density of healthy food outlets	The concentration of healthy food outlets in a given location.
13.	Density of unhealthy food outlets over healthy food within 400 m buffer	The density of unhealthy food outlets over healthy food within 400 from household locations.
14.	Physical Exposure to unhealthy food over healthy food	The extent to which individuals are exposed to unhealthy food outlets compared to healthy food outlets.
15.	Average operating hours for unhealthy food outlets	The typical hours of operation for unhealthy food outlets.
16.	Average operating hours for healthy food outlets	The typical hours of operation for healthy food outlets.
17.	Preference for out of home consumption	A person’s preference for eating meals outside of the home, rather than preparing and eating meals at home.
18.	Cooking skills	The ability to prepare and cook food.
19.	Time available for meal preparation	The amount of time a person has available to dedicate to preparing meals.
20.	Eating out budget	The amount of money that a person or household allocates towards eating meals outside of the home, such as at restaurants, fast food chains, cafes, or other food establishments.
21.	Household income	Household income refers to the total amount of money earned by all members of a household
22.	Customer buying power for healthy food	The ability of a consumer to purchase goods and services.
23.	Actual exposure to unhealthy food outlets over healthy food outlets	The number of unhealthy food establishments affordable that an individual is exposed to compared to healthy food establishments.
24.	Cultural norms for healthy eating	The values, beliefs, and behaviours that are considered acceptable within a particular cultural group with regard to food and nutrition.
25.	Peer influence for unhealthy food	The impact that the eating behaviours and habits of one’s social network can have on one’s eating habits and behaviours.
26.	Consume unhealthy meals over healthy meals	Pattern of choosing to eat meals that are high in calories, fat, salt, and sugar over those that are nutritious, and minimally processed.
27.	Food preference for unhealthy food	A person’s desire for foods that are high in calories, fat, salt, and sugar and low in nutrients.
28.	Age	Age is a demographic characteristic
29.	Active lifestyle	A person’s lifestyle includes pattern of physical activity and exercise in daily life.
30.	Palatability of food offering	The degree of satisfaction that a person experiences when consuming a particular food or meal.
31.	Education	Education is a demographic characteristic
32.	Food and nutrition knowledge	A person’s understanding and awareness of the relationship between food, diet, and health.
33.	Comprehensibility of nutritional information	Ease with which a person can understand and make use of information about the nutritional content of food.
34.	Awareness campaign for healthy food	Efforts to educate and inform people about the importance of consuming a healthy diet.
35.	Food label quality	The accuracy, completeness, and clarity of information provided on the food label or packaging.
36.	Nutritional quality of food and drinks	The balance of nutrients, vitamins, and minerals that are contained in a particular food or drink.
37.	Price of healthy food	The cost of purchasing healthy food for the consumer.
38.	Price of unhealthy food	The cost of purchasing unhealthy food for the consumer.
39.	Ratio unhealthy food price over healthy food price	The comparison of the cost of purchasing unhealthy foods with the cost of purchasing healthy foods.
40.	Profit unhealthy food outlets	The financial gain realized by businesses that sell unhealthy food.
41.	Development of unhealthy food over healthy food	The quantity of unhealthy food that is produced and manufactured compared to the quantity of healthy food.
42.	Cost of production	Expenditures incurred to obtain a product.
43.	Industry desire for profit	Industry intent to achieve a monetary gain.
44.	Promotion of unhealthy food	Activities used to inform customers about the product to create awareness, increase demand, and drive sales.
45.	Exposure to ads for unhealthy option	The extent to which individuals are exposed to advertising campaigns about unhealthy food options.
46.	Brand reputation for unhealthy food	How a brand is perceived in public.
47.	Reward system sensibility	How sensitive are individuals to incentives offered.
48.	Brand loyalty for unhealthy food	A person’s dedication towards a particular brand.

As we previously described, at this stage no attempt was to make the CLD perfect as the goal was to enrich the initial model through the knowledge elicitation process. However, a core positive feedback loop from “exposure to unhealthy food” to “consumption of unhealthy food” to “attractiveness for unhealthy food retailers” and back to “exposure of unhealthy food” (through the number and density of food outlets) was straightforward to identify in the CLD due to the graphical representation of the variables and arrows (Fig 6). This was done on purpose, to indirectly remind participants of feedback processes within the model. Next, we present how each section of the preliminary model was refined through the knowledge elicitation process and represented in the final model.

#### Section I: Exposure to HFSS food

Section I of the questionnaire and workbook included statements that captured the relationship between the dependent variable “Exposure to HFSS foods” and other independent variables. Starting from the upper section of the preliminary model, we present below the changes made to this section during the knowledge elicitation process. Based on the data collected from participants, we identified the variable "attractiveness for unhealthy food retailers" as needing redefinition. This variable was found to be influenced by several factors, including the socioeconomic status (SES) of the local area, population size, and the cost of rent for commercial space. Initially, in the preliminary model, the aesthetic value of the neighbourhood was also considered as a contributing factor to the “attractiveness for unhealthy food retailers" variable. However, further analysis revealed that this variable is a composite variable that is represented indirectly by other variables such as rent and SES. As a result of this finding, the variable was removed from the CLD since its influence on the system can be accounted for through the relationships with other relevant variables.

Moreover, the variable “attractiveness for unhealthy food retailers” was considered by participants to affect the presence of both types of food retailers in the neighbourhood which are represented by the variables “Number of fast-food”, “Number of convenience stores”, “Number of express markets”, and “Number of restaurants”. These variables were found to directly affect “density”, which was calculated by considering the number of these specific stores relative to the overall number of all types of stores in the area.

The variable "exposure to unhealthy food" was assessed based on the density of food outlets, the operating hours of the food retailers and the price of healthy food alternatives (“Price of products offered” variable). The questionnaire results indicated that both the density and price of a product have an impact on "exposure to unhealthy food”. However, the influence of the price of a healthy alternative on exposure was not entirely clear. Its effect seemed to depend on other factors such as SES and household income and seemed to influence more purchase and consumption of unhealthy food rather than exposure to unhealthy food. Further analysis was conducted to better understand how these factors interact and influence exposure to unhealthy food and in the workshop version of the model, the prices of healthy and unhealthy food were included as variables that influence the purchase and consumption of unhealthy food.

**Fig 7:**
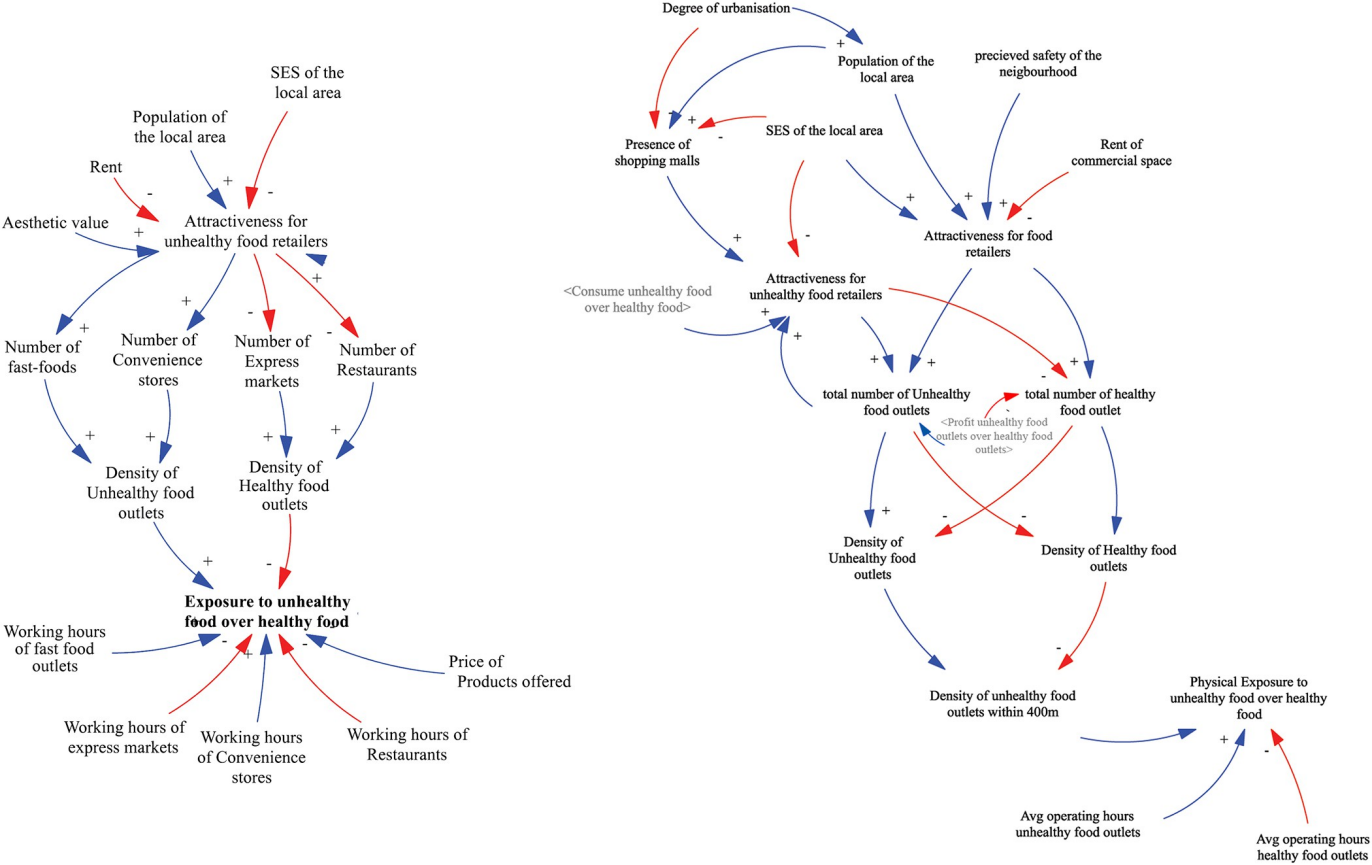
Changes in section I of the CLD. Left side represents the preliminary model as presented in questionnaire. Right side represents the model as presented in the workbook, workshop, and the final model. Blue line represents a positive link, indicating a move in the same direction. Red line represents a negative link, indicating a move in different directions. Greyed variables represent shadow variables that are defined elsewhere in the model.

As a result of these findings, the “exposure to unhealthy food over healthy food” needed to be better defined. Most participants indicated that the presence of unhealthy food retailers in the immediate environment is not a good way to define this variable even though the increased density of food outlets drives the tendency for snacking or ‘grazing’ which then may increase the preference to consume the type of food on offer. However, this way of defining the variable does not refer to the actual exposure. For instance, if an individual cannot afford the type of food they are exposed to, they are unable to purchase and consume it. Hence, the findings suggested a need to differentiate between physical exposure to unhealthy food over healthy food, which considers the density of food outlets within a 400m distance from individuals’ location and their operating hours; and the actual exposure, which considers physical access and financial access to healthy food. The findings illustrate the changes made to the model during each stage of the knowledge elicitation process, as depicted in Fig 7.

#### Section II: Consumption of HFSS foods

Section II included statements that captured the relationship between the dependent variable “Consumption of HFSS foods” and other independent variables. Starting from the right side of the preliminary model, we present below the changes made to this section during the knowledge elicitation process.

In the preliminary model, the variable "food preference for unhealthy food" was initially believed to be affected by age, the cost of healthy foods, cultural norms for healthy food, and the development of unhealthy food products. However, through the knowledge elicitation process, it was revealed that price does not directly impact preference, and certain factors like palatability and lifestyle were not initially considered. Hence, the variable "food preference for unhealthy food" was found to be influenced by age, household cultural norms, lifestyle, palatability of food, and the actual purchase and consumption of unhealthy food. Participants considered household cultural norms a very important factor affecting an individuals’ preference for consuming unhealthy food. Participants also recognised that the consumption of foods high in fat, salt, and sugar, furthermore, increases individuals’ preference for such foods due to their high palatability and because the body becomes accustomed leading to more cravings.

In the workbook’s causal loop diagram, the link from the variable "Consumption unhealthy food over healthy" to the variable "Food preference for unhealthy food" didn’t have any delay. However, during the workshop, a participant pointed out that a delay should be present in this representation. The participant highlighted that food preference can develop gradually over time through repeated consumption of a particular food. This observation was acknowledged and supported by other participants during the workshop, leading to a consensus that a delay should indeed exist in the model. As a result, the final version of the model incorporated the delay in the link, reflecting the collective agreement and understanding that food preference is influenced by the cumulative effect of repeated consumption experiences.

**Fig 8:**
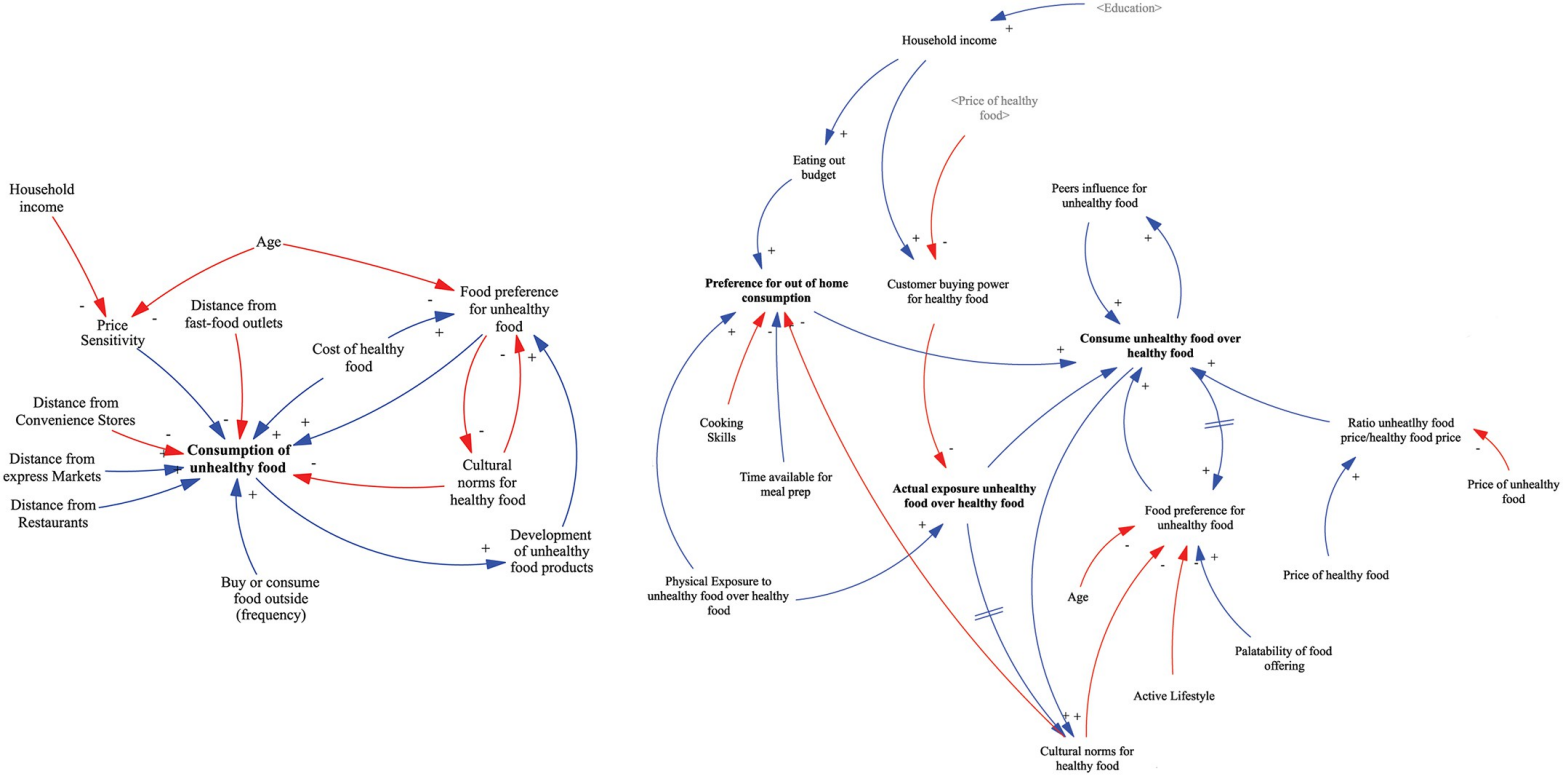
Changes in section II of the CLD. Left side represents the preliminary model as presented in the questionnaire. Right side represents the model as presented in the workshop and the final model. Blue line represents a positive link, indicating a move in the same direction. Red line represents a negative link, indicating a move in different directions. Greyed variables represent shadow variables that are defined elsewhere in the model.

In the preliminary model, consumption was influenced by shop distance, frequency of eating out, price sensitivity, cost of healthy food, food preference, and cultural norms. However, participants found that the consumption of healthy and unhealthy food is influenced by various factors such as the proximity of their place of residence, work, or school to the food retailer, personal food preferences, peer influence, advertising, and price. These findings illustrate the changes made to the model during each stage of the knowledge elicitation process, as depicted in Fig 8.

**Fig 9:**
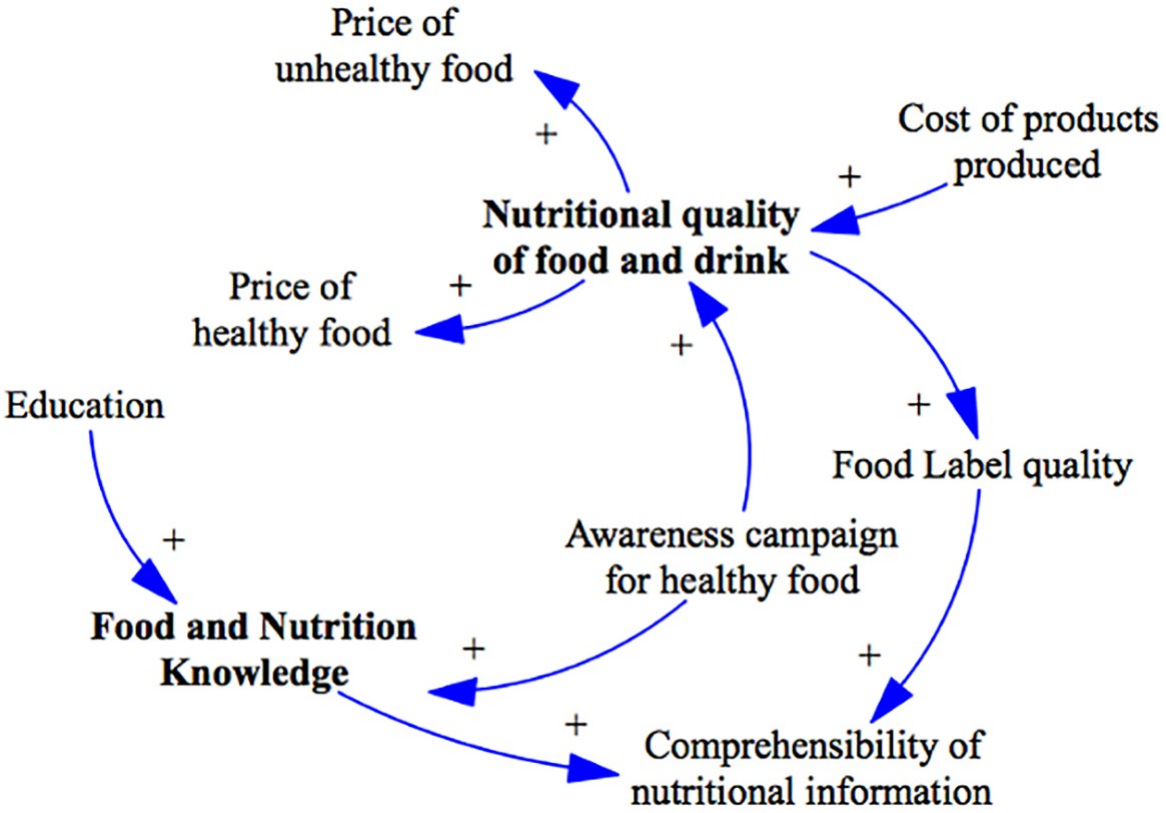
Food and nutrition knowledge system structure. Blue line represents a positive link, indicating a move in the same direction. Red line represents a negative link, indicating a move in different directions.

Results from the questionnaire analysis indicated the need for an additional system structure to be added to the causal loop diagram. This newly identified structure pertains to the food and nutrition knowledge of individuals. The participants emphasized the significance of incorporating this aspect into the model to better understand its impact on the overall system dynamics. Fig 9 shows how this structure was represented in the workbook, workshop and the final model.

The variable "Food and nutrition knowledge" within the model represents the health literacy of individuals, encompassing their ability to understand, evaluate, and apply health information to their daily lives. It was observed that this variable is influenced by education and awareness campaigns focusing on healthy food. While other factors also influence "Food and nutrition knowledge," they were considered to lie outside the model’s scope and were not included in the current representation. The variable "nutritional quality of food and drinks" was incorporated into the model. This variable was found to impact the pricing of the products being sold. It was also observed that awareness campaigns promoting healthy products can affect the nutritional quality variable. Conversely, the variable "nutritional quality of food and drinks" was seen to influence the quality of product labels. When the information presented on food products is of higher quality, it becomes more comprehensible to individuals. Additionally, it was found that individuals’ food and nutrition knowledge also affects their ability to comprehend the information presented on product labels. This structure was validated by participants during the workshop.

**Fig 10:**
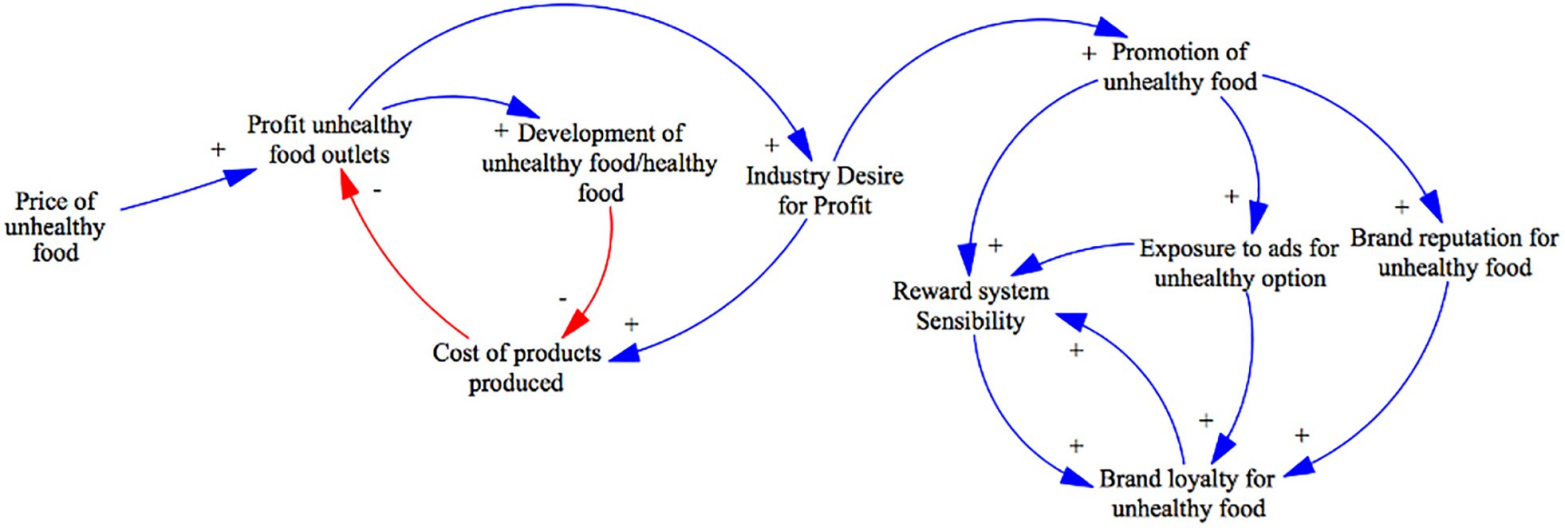
Business perspective system structure. Blue line represents a positive link, indicating a move in the same direction. Red line represents a negative link, indicating a move in different directions.

Furthermore, the results of the questionnaire, showed that approximately 80% of the participants considered advertising and food promotion to be significant factors influencing their decisions to consume and purchase HFSS food. These findings emphasized the substantial impact of advertising on individuals’ food choices, which has been largely supported by literature, and highlight the importance of representing this domain in the system map of the food environment (Fig 10).

In the preliminary model, the business perspective was not taken into account, and only the variable "development of unhealthy food products" was included, which was influenced by the consumption of unhealthy food. The underlying assumption was that an increase in unhealthy food consumption would drive the development of more unhealthy products. This assumption was based on the idea that higher demand for unhealthy food would lead to increased production and consumption of such products. In the refined model, we followed a similar logic and introduced a new variable called "profit." This variable represents the difference between the price of the product and the cost of production. According to our analysis, a more profitable product would lead businesses to allocate more funds to increase their production and outlets. As production scales up, the cost of production is expected to decrease due to economies of scale.

Additionally, the pursuit of higher profits within the industry drives the usage of the marketing mix as a strategic tool. The promotion of unhealthy food plays a significant role in this regard. It leads to increased exposure to unhealthy advertisements, enhances the brand reputation for unhealthy food, and influences individuals’ sensitivity towards rewards schemes implemented by food retailers. These three factors collectively contribute to brand loyalty for unhealthy food. Finally, brand loyalty affects individuals’ sensitivity towards rewards schemes, completing the causal loop within the model. This interconnected framework highlights the influence of the business perspective on the production, marketing, and consumption dynamics of unhealthy food products. This structure was as well validated by participants during the workshop.

#### Section III: Exposure and consumption of HFSS food

**Fig 11:**
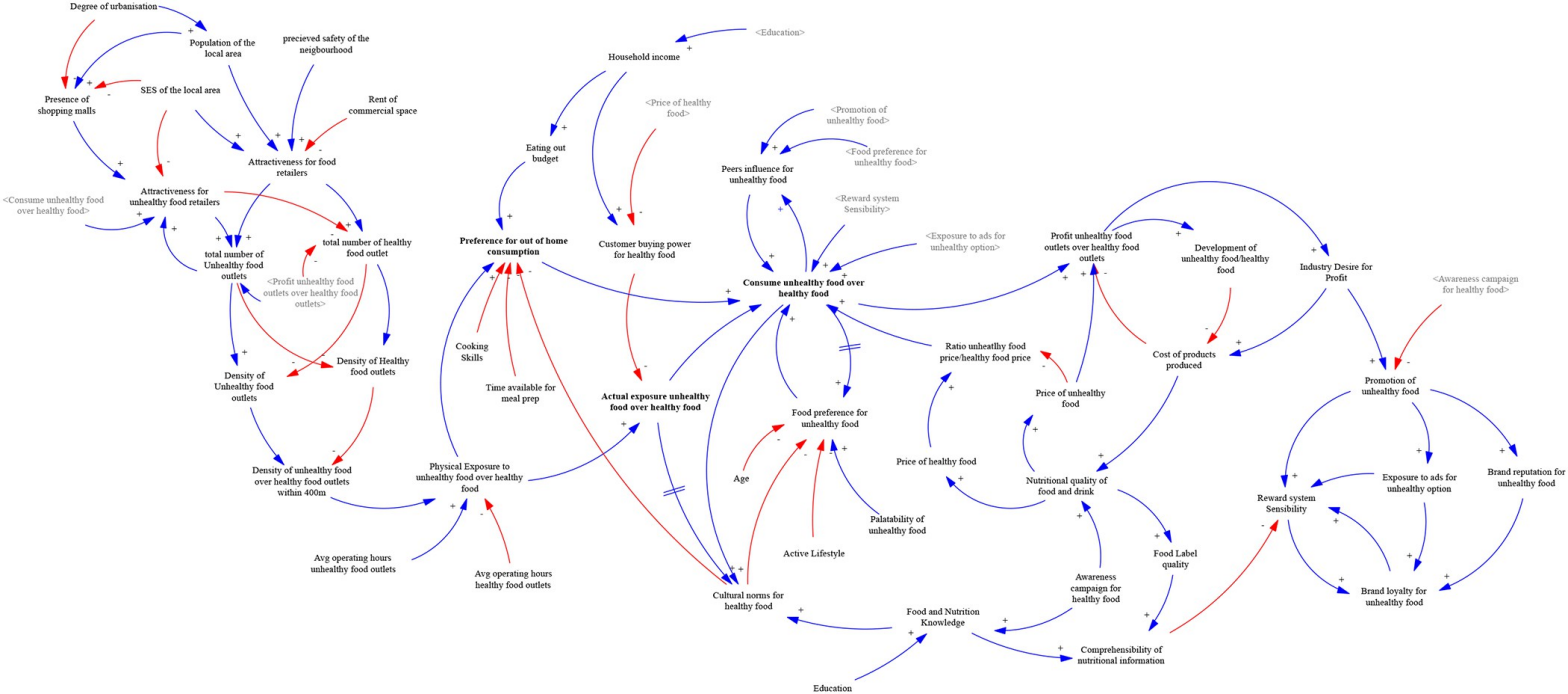
Final conceptual model of the food retail environment. Blue line represents a positive link, indicating a move in the same direction. Red line represents a negative link, indicating a move in different directions. Greyed variables represent shadow variables that are defined elsewhere in the model.

Section III explored the relationship between the two main variables: “exposure to HFSS foods” and “consumption of HFSS foods”. Participants linked both variables positively and they all agreed with the given statement. Exposure is more likely to lead to purchase and consumption of HFSS food if taste and price are fair, if it is conveniently located in your surroundings and socially acceptable. Additionally, exposure is more likely to lead to the purchase and consumption of HFSS food if there are few healthy choices or healthy food alternatives are less tasty, less convenient, more expensive, and normally not consumed by your family and peers. There are also some moderating variables influencing the link between the two main variables like age, SES, household food culture, household size, and working hours. The final model developed after the GMB includes 48 variables in total (Fig 11).

Table 3 presents a summary of the responses to the questionnaire and the workbook (Phase I and II of the knowledge elicitation process).

**Table 3:** Questionnaire and workbook results.

Activity	Aims and description	Results
**Section I: Exposure to HFSS foods**	What variables of the neighbourhood food retail environment are relevant in conceptualising exposure to HFSS foods? How are these variables linked to each other and what are the mechanisms involved (feedback loops)? Validate the new variables, links and structures added to the system (workshop aim).	The section of the preliminary conceptual model was revised and updated. The model was validated by the participants.
Conceptual model review	Participants were presented with the preliminary conceptual map of this section. The relevant CLD was included followed by statements indicating the causal relationship between the dependent variable and other variables. Participants were invited to agree, partially agree, or disagree with the given statements. Next, they were invited to elaborate on their choice.	“Attractiveness for unhealthy food retailers” was reformulated. “Aesthetic value” was a composite variable that was decomposed. A distinction was made between “Physical exposure to HFSS foods” and “Actual exposure to HFSS foods”.
	Participants were asked to add up to three important variables three that affected the main variable, which were not considered in the preliminary model.	The initial conceptual model included 16 variables in this section. The revised model included 18 variables.
Questionnaire model review	The feedback received from the questionnaire was included in this section. The arguments raised in the questionnaire by the participants, according to each statement, were elaborated on and presented in terms of causal links with assigned polarity. Participants were invited to comment, criticise, add, or even cross out links and variables for each statement.	A description of how variables are linked and how they affect the system was sent by participants. Feedback loops were identified within the section. All new reformulations were validated. No new variables were added.
**Section II: Consumption of HFSS foods**	Which variables are responsible for increasing the consumption of HFSS food? How are these variables linked to each other and what are the mechanisms involved (feedback loops)? Validate the new variables, links and structures added to the system (workshop aim).	The section of the preliminary conceptual model was revised and updated. The model was validated by the participants.
Conceptual model review	Participants were presented with the preliminary conceptual map of this section. The relevant CLD was included followed by statements indicating the causal relationship between the dependent variable and other variables. Participants were invited to agree, partially agree, or disagree with the given statements. Next, they were invited to elaborate on their choice.	The “preference for unhealthy food” variable was redefined. “Household cultural norms” was considered a very important factor. Exposure was linked with consumption. The “out of home consumption” variable was redefined. The business point of view was introduced to the model.
	Participants were asked to add up to three important variables three that affected the main variable, which were not considered in the preliminary model.	The initial conceptual model included 13 variables in this section. The revised model included 24 variables, where 15 variables conceptualised the business point of view.
Questionnaire model review	The feedback received from the questionnaire was included in this section. The arguments raised in the questionnaire by the participants, according to each statement, were elaborated on and presented in terms of causal links with assigned polarity. Participants were invited to comment, criticise, add, or even cross out links and variables for each statement.	A description of how variables are linked and how they affect the system was sent by participants. Feedback loops were identified within the section. All new reformulations were validated. No new variables were added.
**Section III: Exposure to HFSS food and Consumption of HFSS foods**	How is exposure to HFSS foods affecting the consumption of HFSS foods?	The variable “Exposure to HFSS foods” was positively linked with the variable “Consumption of HFSS foods”. Feedback loops were identified between both sections of the preliminary conceptual model.
Questionnaire model review	This section aimed to find the incorrect links in the current model. Participants were presented with the “cause and use trees” for each section.	“Cause and use tree” diagrams were validated adding to the strength of the links presented within the model.

Most changes in the preliminary model were implemented after the Questionnaire and Workbook phases. During the workshop, participants focused on validating the existing conceptual model rather than introducing new elements. Another objective was to enhance the model’s visual representation, ensuring its comprehensiveness and readability. This involved clarifying the relationships between existing variables and adjusting the model’s format to improve overall clarity.

#### Representing the key dynamics of the system

**Fig 12:**
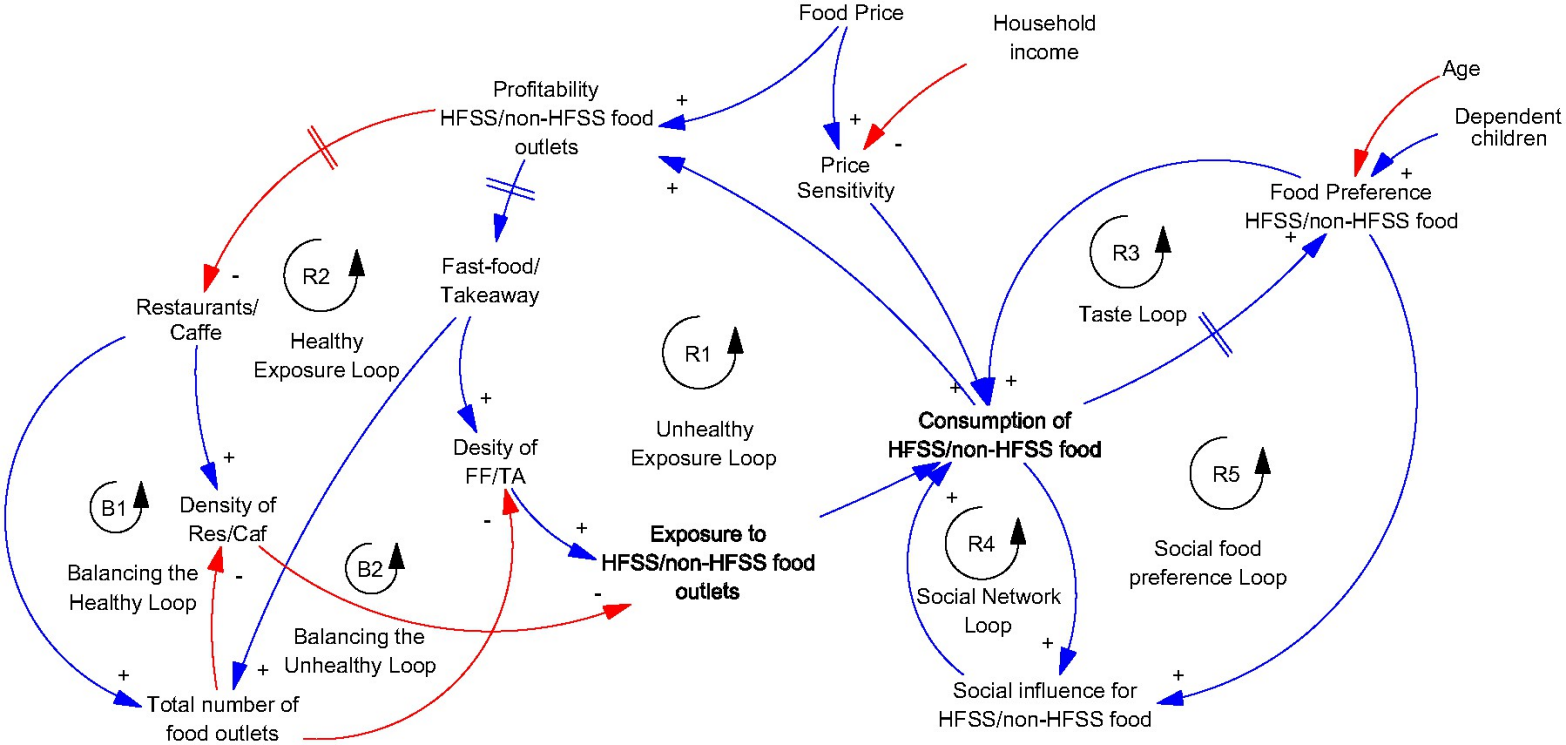
Key feedback loops of the model. Blue line represents a positive link, indicating a move in the same direction. Red line represents a negative link, indicating a move in different directions. Greyed variables represent shadow variables that are defined elsewhere in the model.

The model analysis revealed a total of 76 feedback loops within the model, centred around the variable "Consumption of unhealthy food over healthy food". However not all feedback loops have a substantial impact on the overall system behaviour. Considering the leverage points and the policies suggested by the participants, while keeping in mind the problem under study, we identified the most critical loops of the systems (Fig 12).

The model presented in Fig 12 shows nine feedback loops in total. These feedback loops showcase the complex interplay between various factors within the model that contribute to the pattern of unhealthy food consumption.

The system maps reveal that the consumption of unhealthy food over healthy food is influenced by several factors, including social influence, food preference, price sensitivity, and exposure to unhealthy food environments. Upon analysing the model further, we identified five important reinforcing feedback loops and two balancing loops. The primary feedback loop we observed is the "unhealthy exposure loop" linking exposure to unhealthy food environments with increased consumption of unhealthy food. This loop suggests that the more we are exposed to such environments, the higher the likelihood of consuming unhealthy food, leading to a further increase in exposure over time due to the rising number of unhealthy food outlets in the area. Similarly, the "healthy exposure loop" operates from the perspective of restaurants, reinforcing the relationship between exposure to healthy food environments and the consumption of healthy food options.

The "social network loop" represents how our social networks influence our decisions to purchase and consume unhealthy food. As we consume more unhealthy food, we, in turn, influence our network to do the same, leading to reciprocal effects. The "taste loop" demonstrates how food preferences impact our purchase and consumption choices, affecting the overall consumption patterns. Furthermore, the "social food preference loop" indicates that social influence not only directly impacts our consumption decisions but also influences our food preferences. Additionally, we identified two balancing loops, namely the "Balancing the healthy loop" and "Balancing the unhealthy loop". These loops function to balance the "healthy exposure loop" and "unhealthy exposure loop" by adjusting the total number of food outlets in the environment, thus impacting the density of both healthy and unhealthy food retailers.

Overall, our analysis of these feedback loops provides valuable insights into the dynamics of the system and highlights the interplay of various factors shaping individuals’ food consumption behaviours. Simulation modelling will be utilized to test the significance and impact of these feedback loops on the purchase and consumption of unhealthy food.

Hence, this structure will serve as the foundation for developing an agent-based model of the local food retail environment. The agent-based model captures the dynamic interactions between individuals and their environment, illustrating how both entities adapt and evolve over time. The ABM allows for the recording and analysis of individual agents’ behaviours and decisions within the system, as well as the collective impact of those decisions on the overall system. Understanding these feedback mechanisms can guide targeted interventions and policy measures to promote healthier food choices and environments.

## Discussion

In this paper, we present the results of an online group model building (GMB) process used to generate a conceptual model of a local food retail environment that drives the purchase and consumption of meals that are high in fat, salt, sugar, and calories. The GMB process elicited the mental models of researchers, policymakers, and practitioners working in the field.

This study presents a rigorous, systematic approach to building causal loop diagrams of the local food retail environment in an online rather than in-person setting. We consolidated previous information and data on urban food environments to develop a comprehensive system map of the food retail environment, including the factors that drive the choice to buy meals high in fat, salt, sugar, and calories. The GMB process offers a unique approach to engage policy-makers in creating a shared understanding of the problem and the system, and take ownership of the model which in turn leads to high commitment in terms of policy application [22]. In addition, the process created an opportunity for researchers and practitioners to gain a hands-on understanding of the fundamental techniques and concepts that drive systems-thinking approaches. This was evidenced by participants, gaining a greater comprehension of the elements of the CLD, and was shown by the way they navigated the model as we moved from the questionnaire to the workbook and from the workbook to the workshop. Overall, the participants gained a good understanding of the problem through a systems lens.

A systems lens helped our participants to understand the problem in a holistic and interconnected way, which during the knowledge elicitation process resulted in the generation of new variables that weren’t captured, and new feedback loops were added to the preliminary conceptual model. During the workshop discussion, when policies were suggested, participants took into consideration the dynamics and the changing nature of the system. We believe that by considering the broader context, interdependencies and feedback loops of the system, participants were able to suggest more informed, comprehensive, and sustainable solutions.

In literature, a consensus exists on the food environment playing a significant role in shaping food choices and diet quality and Group Model Building has been often used to explore this system. Overall, studies using GMB have emphasised the need for interventions that address the broader food environment, including improving access to healthy food options and reducing exposure to unhealthy food environments [23, 24]. Our study presents similar findings. Besides building a map of the food retail environment and identifying the main factors that drive individual’s food choices, such as price of food, exposure to the retail environment, food preference and social network influence; our study contributes to the systems dynamics literature by adapting GMB in an online setting. To our knowledge, only three studies have done this, one paper reflects on moving GMB workshops in an online setting [25], another one uses GBM online to explore innovations towards renewable energy in Norway [26], and the last one uses GMB to frame the commercial determinants of dietary behaviours in adolescents in the UK [27].

Overall, undertaking the GMB online was positive, demonstrating that it retained utility compared to the ‘gold-standard’ in-person approach. This process allowed the inclusion of participants from different cities and countries which would have not been possible otherwise. In terms of participation, expectations were that the online session would increase the number of people attending as the physical presence was not required, but this didn’t result true for our sample. This doesn’t mean that this is the rule for any other sample as online workshop eliminate the need for travel, reducing time and cost expenses, and allowing participants to work in their preferred environment.

However, this process doesn’t come without its limitations. One important thing to keep in mind is that technical issues are very common and can affect the flow of the session. For instance, during our workshop, due to poor internet connectivity, a few participants were unable to engage with the whiteboard. During this time, the facilitators must continue with the agenda, while another team member can assist participants virtually to resolve the issue or, if not, pass on their messages. Another thing that might hinder this process is the lack of face-to-face interactions. During our workshop, for ethical considerations, the participants were required to have their cameras off which limited opportunities for nonverbal communication and for building trust among the participants. In terms of delivering tasks, it was challenging to ensure that participants met their deadlines and remained engaged, causing significant delays throughout the process.

As for the facilitation process, online sessions can be more challenging to manage as it can be tricky to have participants attention while controlling and advancing the agenda. Also, we noticed that participants were more hesitant to express their views. Several calls for volunteers, were made during the workshop, but to no avail. To address this issue, the facilitator, keeping in mind the information collected from the questionnaire and workshop, had to proactively ask specific people to contribute. In this regard, other researchers have reached the same conclusion [26]. To this end, we believe that the choice between a physical or a virtual Group Model Building should be based on the objectives of the session, the resources at hand, and the participant’s requirements and preferences. Both methods offer advantages and drawbacks, and in some situations, a combination of both may be the most suitable choice.

The GMB process started with a preliminary conceptual model. Choosing a preliminary model instead of building from scratch facilitated the model building process and allowed it to progress faster as the participants had a starting point for discussion. The preliminary model provided context and allowed participants to prioritise and focus the discussion which might lead to a better outcome. In addition, it helped to clarify how a system is conceived and represented in a CLD, and this was particularly helpful for participants with limited previous knowledge of system dynamics and model building. From the modeller’s side, the preliminary model helped in drafting a comprehensive and coherent questionnaire and workbook. Also, it facilitated the communication between the modeller and the participants, which in an online environment is a challenging task when it comes to sharing systems concepts, models objectives and goals.

Nevertheless, a preliminary model can introduce the modellers’ bias to the GMB and limit the knowledge elicitation process as some participants may be resistant to changing what is there. In this regard, in our study, the modeller kept the model as simple as possible and incomplete and dedicated a good part of the questionnaire to validate what was in the model and identify what was missing from it. Lastly, the preliminary model limits the flexibility of the participants and might even put in jeopardy their creativity.

Constructing the models between the stages of the online knowledge elicitation process was less straightforward compared to an in-person GMB setting. Research literature was consulted to generate the necessary information. Statements about the validity of such connections were included in the following stage of the knowledge elicitation process. In addition, where the results from the questionnaire were inconclusive or uninterpretable, clarifying statements were also included in the workbook so the model building process for those variables could be done in the next iteration.

Although the questionnaire and workbook provided the modeller with information on how different independent variables affected the two main dependent variables specified in the model, and on the link between the two variables, this information was not always sufficient to update the model. Hence, the modeller had to fill any gaps between these dependent and independent variables where the participants’ responses did not provide sufficient information. Although the modeller sought to minimise this process, it still carries potential risks. The modeller must be very careful when filling in the gaps as the information might be inaccurate or not representative of real-world systems, leading to incorrect conclusions. In addition, the process may be affected by the modeller’s own biases and perspectives influencing the information he chooses to fill in. For these reasons, any variable added to the model, was later validated by the participants of the GMB.

Pilot testing of the questionnaire and workbook was effective, especially for those participants not familiar with system dynamics. Expertise in the methodology, occasionally means that the researcher becomes too technical. Hence, piloting helps ensure that the material is clear and understandable for a lay user.

Lastly, one occurring challenge faced when working with public health experts was the discussion on causality versus association. As a considerable number of studies in the field relies on cross-sectional methods, and even though there are a good number of employed multilevel models [28], considerable debate still exists on whether the associations observed reflect causal processes [29]. However, as we are trying to capture mental models, we cannot avoid discussing causal links, as they are a central feature of mental models. In system dynamics, we assume that the world is such that causal links between variables may be specified to explain events or develop policies. However, the elicitation process always yields a collection of causal attributions, or first hypotheses about a system’s structure, which according to Sterman [13] must then be tested.

## Conclusion

This study presents a participatory modelling approach with population health experts. The conceptual model built illustrates the complexity of risk factors and determinants responsible for inequalities in healthy eating. According to practitioners and academics working with food systems, exposure to an unhealthy food retail environment is positively linked with HFSS meal consumption in the neighbourhood. The process was finalised with a conceptual CLD of the urban food environment which will serve as a starting point for building a spatial agent-based model in the future. By describing the followed process and by sharing our experience and insights in applying system approaches in the field of public health we hope to advocate more for its usefulness and encourage public health experts to benefit from the methodology. In a next stage, an Agent-Based model will be developed based on the system map presented in this paper. The model aims to understand how exposure to the neighbourhood food retail environment impacts the consumption of unhealthy food outside of home and simulate the impact of policy interventions aimed at encouraging healthier dietary behaviours in the neighbourhood food retail environment.
